# Statins induce monocytic differentiation in acute myeloid leukemia cells through the KLF4/DPYSL2A axis

**DOI:** 10.1002/2211-5463.70104

**Published:** 2025-08-08

**Authors:** Mina Noura, Kota Shoji, Michidai Nobe, Moe Ishikawa, Miu Tanaka, Akiko Okayama, Souichi Adachi, Hidemasa Matsuo

**Affiliations:** ^1^ Division of Cellular and Genetic Sciences, Department of Integrated Health Sciences Nagoya University Graduate School of Medicine Japan; ^2^ Human Health Sciences, Graduate School of Medicine Kyoto University Japan; ^3^ Shiga General Hospital Moriyama Japan

**Keywords:** DPYSL2A, KLF4, leukemia, statins cell differentiation

## Abstract

Acute myeloid leukemia (AML) is a bone marrow malignancy characterized by arrested early‐stage hematopoietic precursor development. Differentiation therapy, which induces terminal differentiation of immature leukemic cells, is less toxic than standard intensive chemotherapy and a promising treatment strategy for AML. Despite the success of all‐trans retinoic acid and arsenic trioxide in treating acute promyelocytic leukemia (APL), effective differentiation therapy for non‐APL AML has not been established. We previously demonstrated that dihydropyrimidinase‐like 2A (DPYSL2A) is crucial for the monocytic differentiation of AML cells. In this study, analysis using the Comparative Toxicogenomics Database identified statins, which are well‐known cholesterol‐lowering drugs, as potential compounds that upregulate *DPYSL2A* expression in a Krüppel‐like factor 4 (KLF4)‐dependent manner. Most of the tested statins promoted the monocytic differentiation of non‐APL AML cells, leading to rapid apoptosis. The statin‐induced effects were reversed by mevalonate (MVA) supplementation, indicating dependence on MVA pathway inhibition. Furthermore, the inhibition of protein farnesylation, a downstream process of the MVA pathway, mimicked the statin‐induced effects, suggesting that farnesylation suppression is essential for statin‐induced *KLF4/DPYSL2A* expression and monocytic differentiation. These findings may help develop more effective differentiation therapies for patients with non‐APL AML.

## Graphical abstract

Statins, identified via the Comparative Toxicogenomics Database, promote monocytic differentiation and apoptosis in non‐APL AML cells by upregulating DPYSL2A through a KLF4‐dependent pathway. Mevalonate supplementation reversed these effects, indicating involvement of the mevalonate pathway. Targeting the KLF4/DPYSL2A axis via statin treatment may offer a novel strategy for differentiation therapy in non‐APL AML.

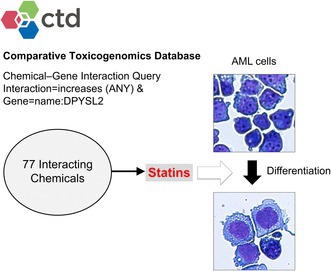

**Abbreviations**
AML: acute myeloid leukemia
ANOVA: analysis of variance
APL: acute promyelocytic leukemia
ATO: arsenic trioxide
CTD: comparative toxicogenomics database
DPYSL2A: dihydropyrimidinase‐like 2A
FBS: fetal bovine serum
HMGCR: HMG‐CoA reductase
KLF4: Krüppel‐like factor 4
MVA: mevalonate
PS: penicillin/streptomycin

Acute myeloid leukemia (AML) is a malignant hematopoietic disorder characterized by the arrest of differentiation and accumulation of immature myeloid progenitors in the bone marrow [1]. AML occurs across all age groups, but its incidence increases significantly with age [2]. In older adults, treatment decisions are influenced by their physical condition. Medically fit patients are recommended to receive intensive chemotherapy using a combination of an anthracycline and standard‐dose cytarabine. Those with poor physical function or high‐risk disease are often treated with supportive care or less intensive chemotherapy, such as hypomethylating agents or low‐dose cytarabine [3]. Due to the high toxicity of intensive chemotherapy, over half of older adults with AML do not receive it as an initial treatment [3], representing an unmet need.

Differentiation therapy is a potentially effective therapeutic strategy with relatively low cytotoxicity that induces the terminal differentiation of immature leukemic cells. Notably, the introduction of all‐trans retinoic acid and arsenic trioxide (ATO) significantly improved the clinical outcomes for acute promyelocytic leukemia (APL), benefiting not only younger patients but also older adults [4]. However, no effective differentiation therapy has been established for patients with non‐APL AML subtypes.

We previously reported that Krüppel‐like factor 4 (KLF4) directly upregulates dihydropyrimidinase‐like 2A (DPYSL2A) expression and potently induces the monocytic differentiation of AML cells [5]. Furthermore, we identified the antihelminthic drugs albendazole and parbendazole as KLF4‐inducing compounds that promote the monocytic differentiation of non‐APL AML cells via the KLF4/DPYSL2A axis [6, 7]. Thus, given the crucial role of DPYSL2A in monocytic differentiation [5], this study aimed to identify compounds that promote monocytic differentiation by inducing DPYSL2A expression through KLF4‐dependent or KLF4‐independent mechanisms.

Comparative Toxicogenomics Database (CTD) is a curated database that facilitates understanding of the effects of environmental chemicals on human health [8]. In addition, it can be used to explore how various compounds alter gene expression profiles across different cell types. Therefore, we utilized this database to identify compounds that upregulate DPYSL2A expression and potentially drive monocytic differentiation. Through CTD screening, we identified statins, HMG‐CoA reductase (HMGCR) inhibitors, as potential inducers of DPYSL2A. Most statins activated the KLF4/DPYSL2A axis and induced the monocytic differentiation of AML cells, subsequently leading to apoptosis. Our findings may potentially improve differentiation therapy for patients with non‐APL AML subtypes.

## Methods

### Cell lines

The human AML cell lines THP‐1 (RCB3686; RIKEN BioResource Research Center, Tsukuba, Japan) and HL‐60 (JCRB0085; Japanese Collection of Research Bioresources, Ibaraki, Japan) were cultured in RPMI 1640 medium (Wako, Osaka, Japan) supplemented with 10% fetal bovine serum (FBS; Thermo Fisher Scientific, Waltham, MA, USA) and 1% penicillin/streptomycin (PS; Wako) at 37 °C under 5% CO_2_. HEK293T cells were maintained in Dulbecco modified Eagle medium (Wako) supplemented with 10% FBS and 1% PS in a humidified incubator at 37 °C under 5% CO_2_.

### Identification of chemicals that interact with DPYSL2A

Curated chemical–gene interactions data were retrieved from the Comparative Toxicogenomics Database (CTD) [8], MDI Biological Laboratory, Salisbury Cove, Maine, and NC State University, Raleigh, North Carolina. World Wide Web (URL: https://ctdbase.org/). [data retrieval: Feb. 2025]. To identify chemicals potentially interacting with DPYSL2A, we used the following parameters: Chemical–gene interaction = increases (ANY) and Gene = DPYSL2.

### Reagents

Atorvastatin (HY‐B0589), fluvastatin sodium (HY‐14664A), lovastatin (HY‐N0504), mevastatin (HY‐17408), pravastatin sodium (HY‐B0165A), and FTI‐277 (HY‐15872A) were purchased from MedChemExpress (Monmouth Junction, NJ, USA). D,L‐mevalonic acid lactone was obtained from Toronto Research Chemicals (Toronto, Ontario, Canada), zaragozic acid A from Cayman Chemical Co. (Ann Arbor, MI, USA), and GGTI‐298 from Aobius Inc. (Gloucester, MA, USA).

### Reverse transcription‐quantitative polymerase chain reaction (RT‐qPCR)

Total RNA was isolated using an RNeasy Mini Kit (Qiagen, Valencia, CA, USA) and reverse‐transcribed using ReverTraAce qPCR RT Master Mix (TOYOBO, Osaka, Japan) to generate cDNA. RT‐qPCR was performed on a Step One Plus Real‐Time PCR System (Applied Biosystems, Foster City, CA, USA), and the amplified products were detected using TB Green Premix Ex Taq II (Tli RNaseH Plus) (Takara Bio, Otsu, Japan). The results were normalized to the expression levels of *GAPDH*, and relative expression levels were calculated using the $2^{-\Delta \Delta C_{t}}$ method. The RT‐qPCR primer sequences were as follows: GAPDH‐F: 5′‐GAAGGTGAAGGTCGGAGTC‐3′, GAPDH‐R: 5′‐GAAGATGGTGATGGGATTTC‐3′, KLF4‐F: 5′‐ACCCTGGGTCTTGAGGAAGT‐3′, KLF4‐R: 5′‐ACGATCGTCTTCCCCTCTTT‐3′, DPYSL2A‐F: 5′‐AAGCCCTGCAGAACATCAAC‐3′, and DPYSL2A‐R: 5′‐TTGCTTGATCAACCCATCTTC‐3′.

### Flow cytometry

The monocytic differentiation of AML cells was assessed using Brilliant Violet 421‐labeled anti‐human CD11b (301324; BioLegend, San Diego, CA, USA) and CD14 (325 628; BioLegend, USA) antibodies on a FACS Canto II Cell Analyzer (BD Biosciences, Bedford, MA, USA). The resultant data were analyzed using flowjo software (BD Biosciences).

### Morphology

Cells were spun onto slides using a StatSpin Cytofuge 2 centrifuge (Beckman Coulter, Brea, CA, USA). Then, each slide underwent Diff–Quik staining (Sysmex, Kobe, Japan), a modified Giemsa staining method.

### Apoptosis assay

After washing the cells in phosphate‐buffered saline, they were suspended in annexin V binding buffer, mixed with 5 μL of annexin V and 7‐AAD (BioLegend), and incubated for 15 min. The cells were then diluted and processed for flow cytometric analysis.

### Statistical analysis

Differences between the control and experimental groups were assessed using a two‐tailed unpaired Student's *t*‐test. The equality of variances in two populations was calculated using the *F*‐test. Statistical significance among three groups was evaluated by one‐way analysis of variance (ANOVA), followed by Tukey's *post hoc* test for multiple comparisons. Data were presented as the mean ± SEM of the values obtained from three independent experiments. Results were considered statistically significant if the *P*‐value was < 0.05.

## Results

### Identification of statins as potential inducers of DPYSL2A expression

**Fig. 1:**
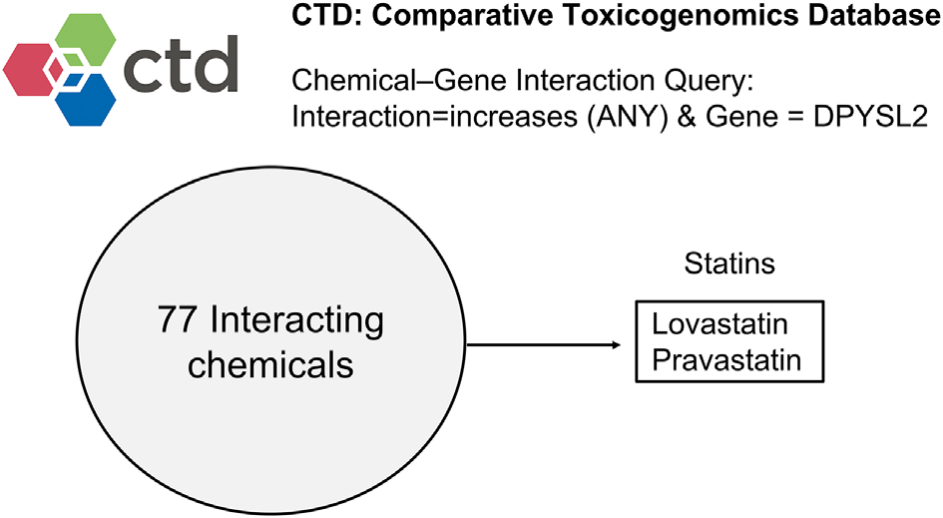
Screening for potential DPYSL2A inducers using the Comparative Toxicogenomics Database (CTD). Compounds associated with increased DPYSL2 expression were identified from the CTD. Among the 77 candidate chemicals, two statins, namely lovastatin and pravastatin, were identified.

To identify potential DPYSL2A inducers, we used the CTD to search for compounds associated with increased DPYSL2 expression (Fig. 1 and Table S1). Among the 77 candidate chemicals, we confirmed the presence of albendazole (Table S1), which stimulates the KLF4/DPYSL2A axis [6]. We also identified two statins, lovastatin and pravastatin, among the candidates. Statins are well‐known cholesterol‐lowering drugs that block HMGCR, the rate‐limiting enzyme of the mevalonate (MVA) pathway [9]. Because statins also exert antileukemic effects on AML cells [10, 11, 12, 13, 14, 15], we focused on their potential to induce monocytic differentiation by increasing DPYSL2A expression.

### Statins induce the monocytic differentiation of AML cells via the KLF4/DPYSL2A axis

**Fig. 2:**
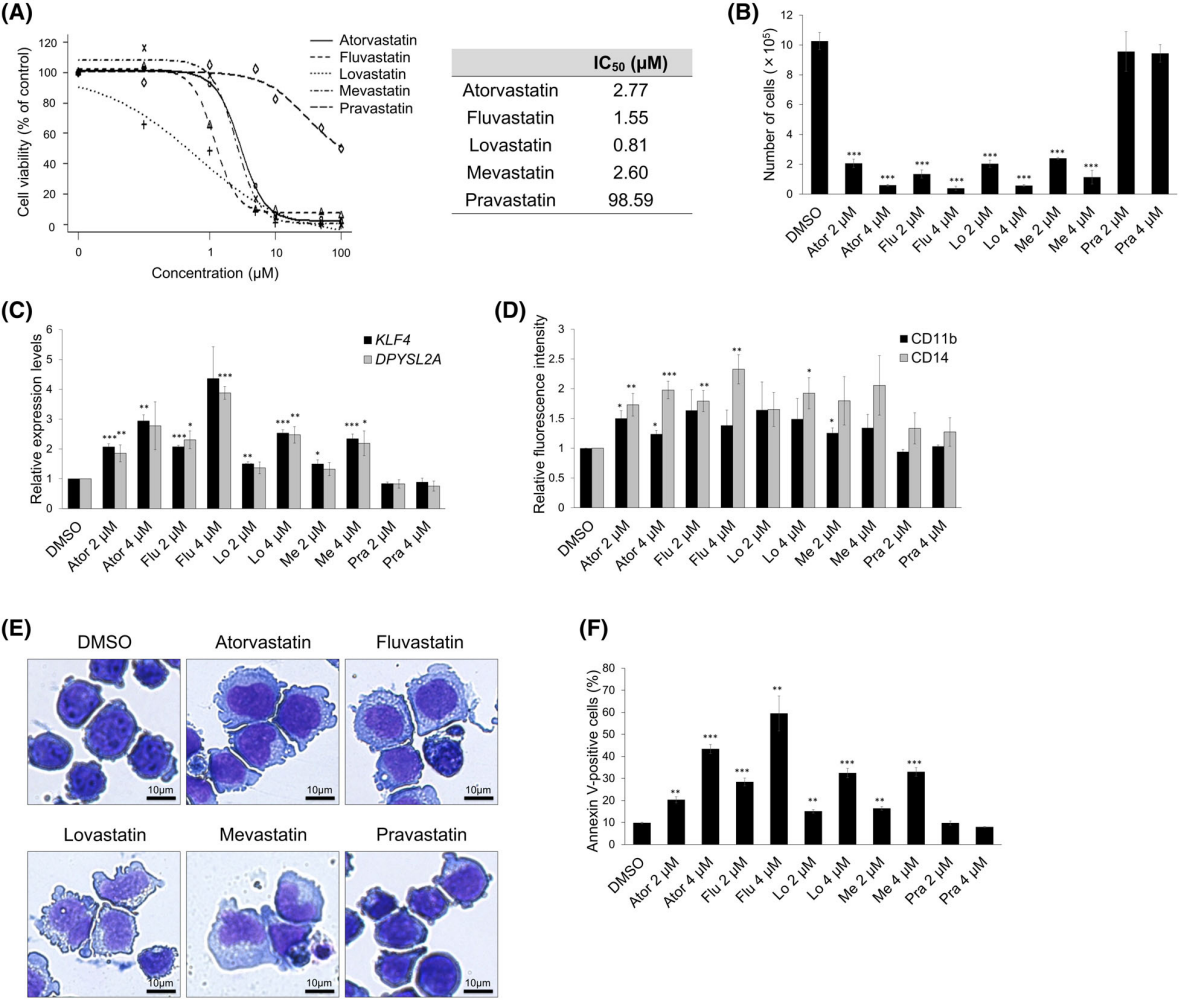
Statins induced monocytic differentiation and suppressed cell proliferation in THP‐1 cells by stimulating the KLF4/DPYSL2A axis. (A) Dose–response curves of the five statins in THP‐1 cells, along with the corresponding IC_50_ values for each statin. The cells were treated with various statin concentrations for 48 h (*n* = 3). (B) Growth inhibition following statin treatment of THP‐1 cells. To assess cell proliferation, 1 × 10^4^ cells were seeded in a six‐well plate and cultured with DMSO or statins (2 or 4 μm). Trypan blue dye exclusion assays were performed after 6 days of treatment (*n* = 4). Abbreviations: Ator, atorvastatin; Flu, fluvastatin; Lo, lovastatin; Me, mevastatin; Pra, pravastatin. (C) Relative mRNA expression levels of *KLF4* and *DPYSL2A* in THP‐1 cells treated as described in (B). Total RNA was then extracted and analyzed by RT‐qPCR. Values were normalized to the *GAPDH* expression levels (*n* = 3). (D) Cell surface expression levels of CD11b and CD14 in THP‐1 cells treated as described in (B) (*n* = 3). (E) Morphological changes in THP‐1 cells treated with DMSO or statins (4 μm) for 6 days. (F) Apoptosis of THP‐1 cells treated as described in (B). The annexin V‐positive cells were scored using flow cytometric analysis (*n* = 3). Data are presented as the mean ± SEM. *P < 0.05, **P < 0.01, ***P < 0.001; two‐tailed Student's *t*‐test.

**Fig. 3:**
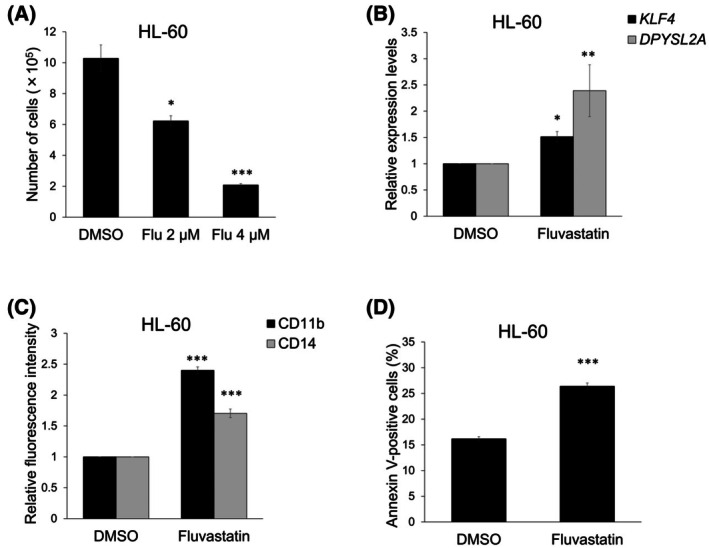
Fluvastatin induces monocytic differentiation in HL‐60 cells via the KLF4/DPYSL2A axis. (A) Growth inhibition by fluvastatin in HL‐60 cells. To assess cell proliferation, 1 × 10^4^ cells were seeded in a six‐well plate and cultured with DMSO or fluvastatin (2 or 4 μm). Trypan blue dye exclusion assays were performed after 6 days of treatment (*n* = 3). (B) Relative mRNA expression levels of *KLF4* and *DPYSL2A* in HL‐60 cells treated with DMSO or 4 μm fluvastatin. Total RNA was then extracted and analyzed by RT‐qPCR. Values were normalized to the *GAPDH* expression levels (*n* = 3). (C) Cell surface expression levels of CD11b and CD14 in HL‐60 cells treated as described in (B) (*n* = 3). (D) Apoptosis in HL‐60 cells treated as described in (B). The annexin V‐positive cells were scored using flow cytometric analysis (*n* = 3). Data are presented as the mean ± SEM. *P < 0.05, **P < 0.01, ***P < 0.001; two‐tailed Student's *t*‐test was used.

Since CTD curation is limited to scientific publications indexed in PubMed [8], we reasoned that our CTD search identified only lovastatin and pravastatin, not because other statins lack effects on DPYSL2A, but because transcriptomic data for those statins have not yet been published or curated in the database. Given that statins share a common core structure and similar pharmacological properties as HMG‐CoA reductase inhibitors, we hypothesized that other statins might also exert comparable biological effects on AML cells. Therefore, we evaluated the effects of five representative statins (atorvastatin, fluvastatin, lovastatin, mevastatin, and pravastatin) on the viability of THP‐1 cells, a human acute monocytic leukemia cell line, and calculated the IC_50_ values for each (Fig. 2A). All statins, except pravastatin, exhibited low IC_50_ values. We then assessed *DPYSL2A* expression at various statin concentrations and found that it was most effectively induced at 2–4 μm (data not shown). Based on these results, we used 2 or 4 μm of each statin for subsequent experiments. All statins, except pravastatin, significantly inhibited cell proliferation in a dose‐dependent manner in THP‐1 cells (Fig. 2B). Additionally, statins increased *KLF4* and *DPYSL2A* expression (Fig. 2C), suggesting that statin treatment upregulated *DPYSL2A* expression through KLF4. Furthermore, statins enhanced the expression of the monocyte differentiation markers CD11b and CD14 (Fig. 2D). Morphological analysis revealed the statin‐induced terminal differentiation of THP‐1 cells into monocytes, characterized by abundant blue‐gray cytoplasm containing fine lilac granules (Fig. 2E). Notably, the differentiated cells underwent rapid apoptosis (Fig. 2F). Because fluvastatin was the most effective inducer of *KLF4* and *DPYSL2A* (Fig. 2C), we treated an additional non‐APL AML cell line (HL‐60) with fluvastatin, resulting in suppressed proliferation in a dose‐dependent manner (Fig. 3A). Furthermore, consistent with the results observed in THP‐1 cells, fluvastatin induced *KLF4* and *DPYSL2A* expression (Fig. 3B), increased CD11b and CD14 levels (Fig. 3C), and promoted apoptosis in HL‐60 cells (Fig. 3D). These observations suggested that statins promoted monocytic differentiation through the KLF4/DPYSL2A axis and exhibited antileukemic activity in non‐APL AML cells.

### Statins promote monocytic differentiation by MVA pathway inhibition

**Fig. 4:**
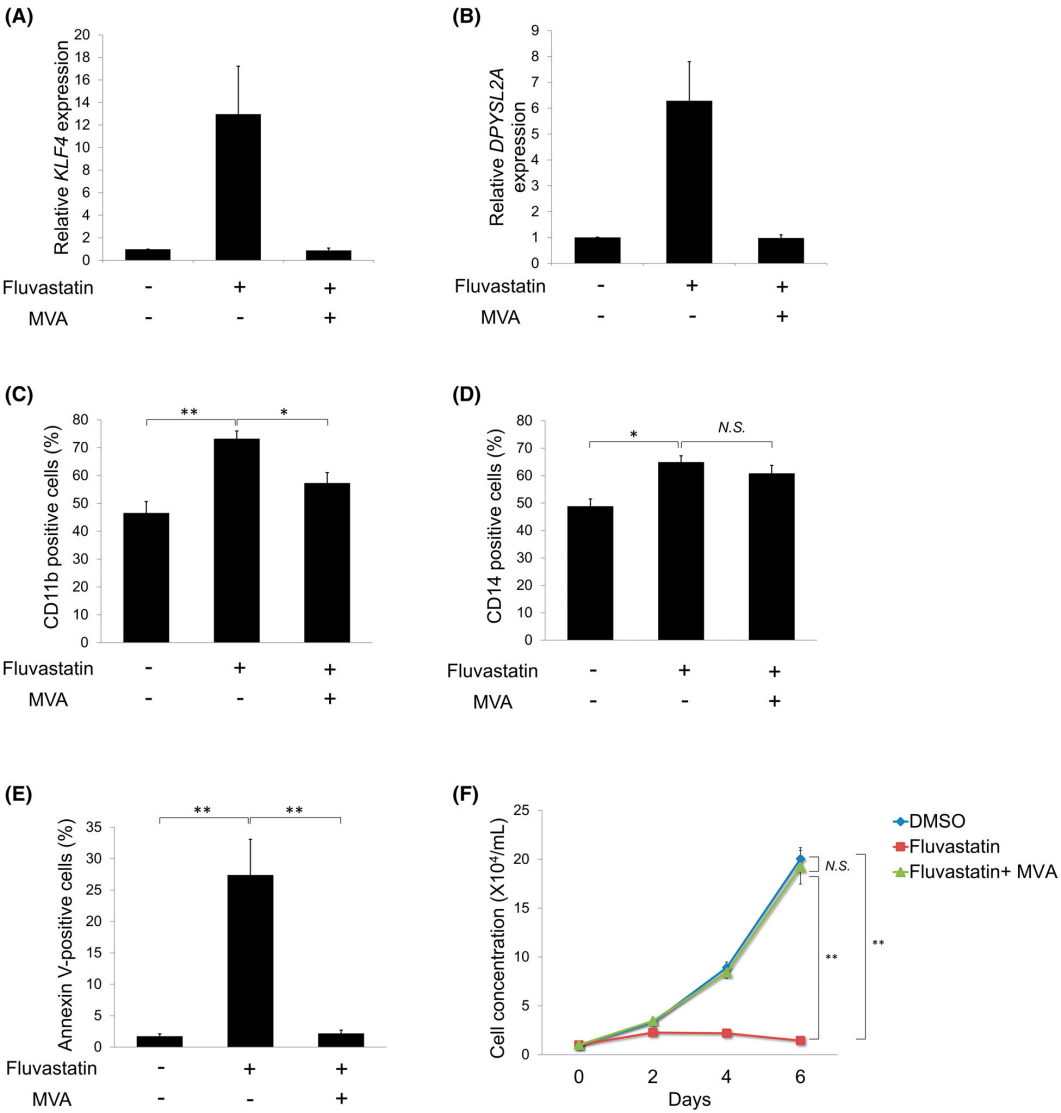
Monocytic differentiation induced by fluvastatin depends on the inhibition of the MVA pathway. (A) Relative mRNA expression levels of *KLF4* in THP‐1 cells. The cells were treated with DMSO, 4 μm fluvastatin alone, or 4 μm fluvastatin in combination with 100 μm MVA for 6 days. Total RNA was then extracted and analyzed by RT‐qPCR. Values were normalized to the *GAPDH* expression levels (*n* = 3). (B) Relative mRNA expression levels of *DPYSL2A* in THP‐1 cells. The cells were treated as described in (A), and then total RNA was extracted and analyzed by RT‐qPCR. Values were normalized to the *GAPDH* expression levels (*n* = 3). (C) Cell surface expression levels of CD11b in THP‐1 cells. The cells were treated as described in (A) and then harvested for flow cytometric analysis (*n* = 5). (D) Cell surface expression levels of CD14 in THP‐1 cells. The cells were treated as described in (A) and then harvested for flow cytometric analysis (*n* = 5). (E) Rescue of THP‐1 cells from fluvastatin‐induced apoptosis following MVA treatment. The cells were treated as described in (A). The annexin V‐positive cells were scored using flow cytometric analysis (*n* = 5). (F) Rescue of THP1 cells from fluvastatin following MVA treatment. To assess cell proliferation, 1 × 10^4^ cells were seeded in a six‐well plate and treated as described in (A). Trypan blue dye exclusion assays were performed every other day (*n* = 5). Data are presented as the mean ± SEM. *P < 0.05, **P < 0.01, N.S., not significant; one‐way analysis of variance (ANOVA), followed by Tukey's *post hoc* test, was used.

The inhibition of HMGCR and the MVA pathway results in statin‐induced cell cycle arrest and apoptosis in cancer cell lines [16]. To determine whether MVA pathway inhibition is essential for statin‐induced monocytic differentiation, we cotreated THP‐1 cells with fluvastatin and MVA. MVA reversed the fluvastatin‐induced expression of *KLF4* and *DPYSL2A* (Fig. 4A,B). Additionally, MVA attenuated fluvastatin‐induced expression of CD11b and CD14 in THP‐1 cells, with a significant reduction observed in CD11b level (Fig. 4C,D). Furthermore, MVA prevented fluvastatin‐induced apoptosis and restored THP‐1 cell growth (Fig. 4E,F). These findings suggested that the statin‐induced monocytic differentiation of AML cells was dependent on MVA pathway inhibition.

**Fig. 5:**
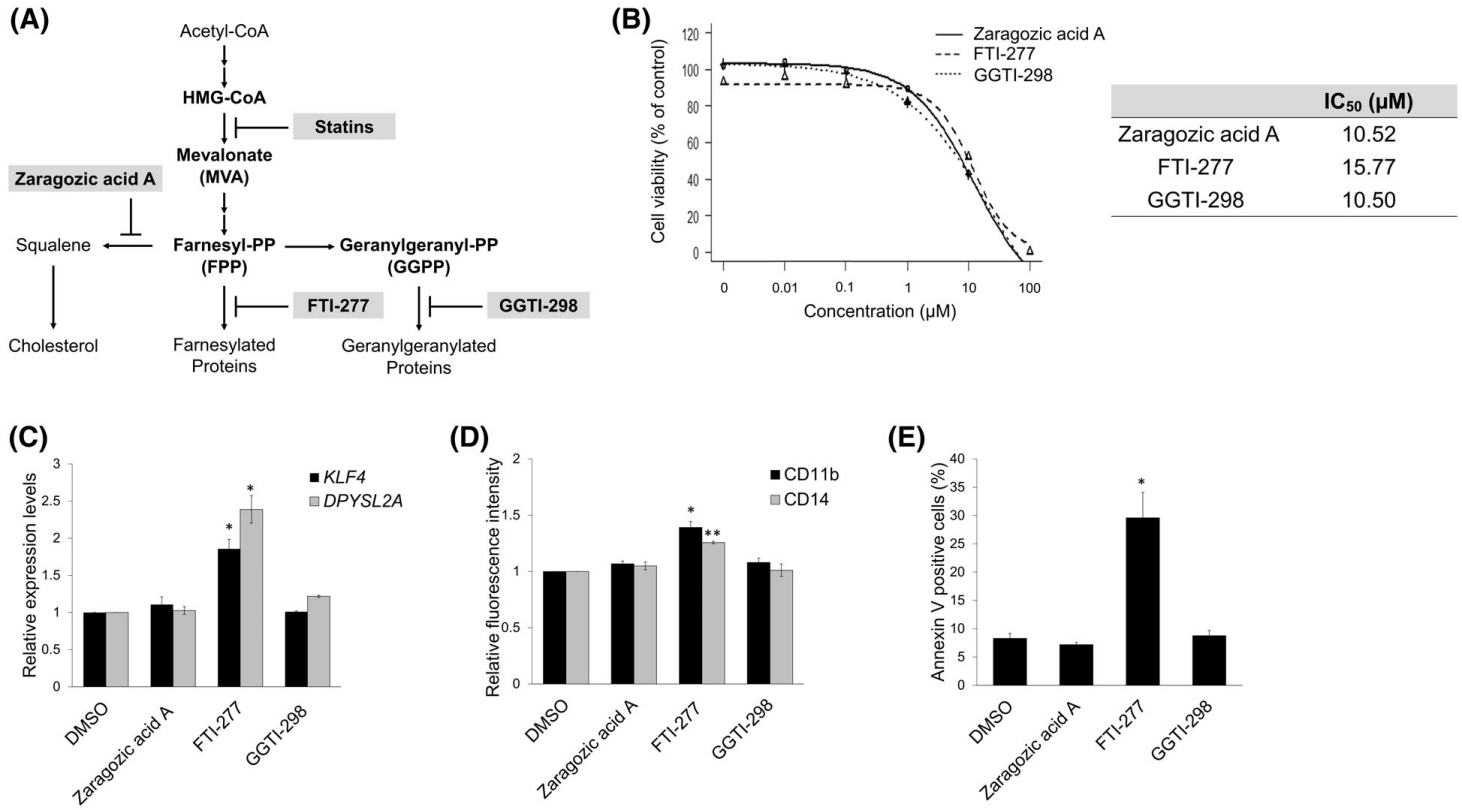
Protein farnesylation inhibition is essential for statin‐induced monocytic differentiation of AML cells. (A) Schematic representation of the MVA pathway. Zaragozic acid A, the farnesyl transferase inhibitor FTI‐277, and the geranylgeranyl transferase I inhibitor GGTI‐298 block the synthesis pathways branching off from FPP in the MVA pathway. (B) Dose–response curves of zaragozic acid A, FTI‐277, and GGTI‐298 in THP‐1 cells, along with their corresponding IC_50_ values. The cells were treated with various statin concentrations for 72 h (*n* = 3). (C) Relative mRNA expression levels of *KLF4* and *DPYSL2A* in THP‐1 cells treated for 72 h with DMSO or 10 μM of zaragozic acid A, FTI‐277, or GGTI‐298, respectively. Total RNA was then extracted and analyzed by RT‐qPCR. Values were normalized to the *GAPDH* expression levels (*n* = 3). (D) Cell surface expression levels of CD11b and CD14 in THP‐1 cells treated as described in (C) (*n* = 3). (E) Apoptosis of THP‐1 cells treated as described in (C). The annexin V‐positive cells were scored using flow cytometric analysis (*n* = 3). Data are presented as the mean ± SEM. *P < 0.05, **P < 0.01; two‐tailed Student's *t*‐test was used.

Statins inhibit downstream products of the MVA pathway (Fig. 5A), including farnesyl pyrophosphate and geranylgeranyl pyrophosphate, which are essential for the isoprenylation of proteins such as Ras and Rho [17]. To identify the key downstream targets of the MVA pathway involved in statin‐induced monocytic differentiation, we compared the effects of squalene synthase inhibitor zaragozic acid A, farnesyl transferase inhibitor FTI‐277, and geranylgeranyl transferase I inhibitor GGTI‐298 on monocytic differentiation. Examination of their impact on AML cell viability revealed IC_50_ values of approximately 10–15 μm (Fig. 5B). Notably, only FTI‐277 treatment upregulated *KLF4* and *DPYSL2A* expression (Fig. 5C), enhanced the levels of CD11b and CD14 (Fig. 5D), and promoted apoptosis in THP‐1 cells (Fig. 5E). These findings suggested that the inhibition of protein farnesylation caused the statin‐induced monocytic differentiation of AML cells.

## Discussion

For decades, statins have been widely recognized as safe and effective treatments for hypercholesterolemia [18, 19]. Besides their cholesterol‐lowering effects, they exhibit antitumor activity in various cancers, including colon, pancreatic, and lung cancers [20, 21, 22]. Epidemiological studies have revealed that patients using statins for cholesterol control have a lower risk of developing certain cancers [23, 24, 25]. Furthermore, their efficacy in hematologic malignancies has been reported. For example, statins suppressed AML cell proliferation *in vitro* and reduced the tumor load in an AML mouse model without damaging normal bone marrow‐derived myeloid progenitor cells [16]. Additionally, clinical trials combining chemotherapy with high‐dose pravastatin in patients with AML have demonstrated promising results [26, 27, 28]. Moreover, atorvastatin and fluvastatin promoted the neutrophilic differentiation of APL cells by activating the c‐Jun NH2‐terminal kinase pathway [10, 29]. Although these findings indicate that statins possess antileukemic properties, their role in the differentiation of non‐APL AML cells remains unelucidated.

In this study, we searched for potential DPYSL2A inducers using the CTD, which led to the identification of statins as candidate drugs that increase DPYSL2A expression. Except for pravastatin, all tested statins induced monocytic terminal differentiation and suppressed cell proliferation in non‐APL AML cells by stimulating the KLF4/DPYSL2A axis. Although pravastatin was among the 77 candidate chemicals (Fig. 1A), it did not induce *DPYSL2A* expression in THP‐1 cells (Fig. 2C). Pravastatin is effective when it is taken up intracellularly by organic anion transporting polypeptide 1B1, which is specifically expressed in the liver [30] and not expressed in blood cells. Thus, it is likely that pravastatin was ineffective in our *in vitro* experiments because we used THP‐1 cells. Pravastatin has been reported to suppress the proliferation of a human hepatoma cell line at a concentration of 50 μm [31]. In contrast, we used much lower concentrations (2 or 4 μm) in our experiments. Thus, higher concentrations of pravastatin might induce monocytic differentiation of AML cells. Further studies are needed to clarify the concentration‐dependent effects of pravastatin.

Accumulating evidence supports the crucial role of the MVA pathway and its rate‐limiting enzyme, HMGCR, in tumor development and maintenance [32, 33]. Increased HMGCR activity has been observed in solid tumors and hematologic malignancies [34, 35, 36, 37, 38, 39]. Moreover, statin‐induced growth inhibition and apoptosis in AML cells have been reported to be reversed by MVA supplementation [40]. Consistent with these findings, we demonstrated that statin‐induced *KLF4/DPYSL2A* expression and monocytic differentiation were dependent on MVA pathway inhibition, suggesting that the MVA pathway plays an essential regulatory role in AML cell proliferation and differentiation. Further investigation of the downstream components of the MVA pathway revealed that the inhibition of protein farnesylation by FTI‐277 mimicked statin‐induced monocytic differentiation (Fig. 5). In contrast, the inhibition of other downstream branches of the MVA pathway did not affect the monocytic differentiation of AML cells (Fig. 5). These findings suggest that the differentiation‐promoting effect of statins on AML cells is primarily driven by inhibiting protein farnesylation. It was reported that the combination of FTI‐277 and ATO effectively induced apoptosis in AML cells [41], suggesting that combining statins with ATO may synergistically promote the monocytic differentiation of AML cells. Further studies are required to identify the specific protein for which farnesylation is essential for AML cell differentiation. Moreover, because previous studies have demonstrated the antileukemic activity of statins in an AML mouse model^17^, the extent to which the mechanisms identified in this study are involved must be verified.

Our findings underscore the clinical potential of statins as potential differentiation‐inducing agents for non‐APL AML. Unlike conventional cytotoxic agents, statins have a favorable safety profile and are already widely used in clinical settings. Therefore, they represent attractive candidates for drug repurposing, particularly for the treatment of older patients who are ineligible for intensive chemotherapy. Importantly, we also found that inhibition of farnesyl transferase mimics the effects of statins, suggesting that this branch of the MVA pathway may represent an additional therapeutic target. Further studies using AML animal models and clinical trials are essential to determine whether statins, either alone or in combination with other agents such as ATO, can effectively induce differentiation and improve clinical outcomes in patients with non‐APL AML.

In conclusion, we demonstrated that statins promote the monocytic differentiation of non‐APL AML cells via the KLF4/DPYSL2A axis. Our findings revealed a novel mechanism for the efficacy of statins on AML cells and may lead to the development of more effective differentiation therapies for patients with non‐APL AML.

## Conflict of interest

The authors declare no conflict of interest.

## Author contributions

HM and M. Noura designed the study and prepared the manuscript. M. Noura, KS, M. Nobe, and AO performed the experiments and analyzed the data. All the other researchers participated in the discussion. All authors approved the final manuscript for submission.

## Supporting information


**Table S1.** List of candidate chemicals related to Fig. 1.

## Data Availability

The data that support the findings of this study are available from the corresponding author upon reasonable request.
