# Supplementary material for: Statins induce monocytic differentiation in acute myeloid leukemia cells through the KLF4/DPYSL2A axis
Source: FEBS Open Bio. 2025 Aug 8;15(12):2021–30. doi: 10.1002/2211-5463.70104 (PMC12667209; doi:10.1002/2211-5463.70104)
Supplement: Supplementary file 1 — Table S1. List of candidate chemicals related to Fig. 1. [file FEB4-15-2021-s001.pdf]

Supplementary Table S1

List of candidate chemicals related to Fig. 1.

Chemical Name	Chemical	CAS RN	Gene Sym	Gene ID	Interaction	Interaction Actions	Reference	Organism Count
1-(4-(6-bromobenzo(1,3)(	C517943		DPYSL2	1808	1-(4-(6-bromobenzo(1,3)dioxol-5-yl)-3a,4,	increases^phosphorylation	1	1
1-Naphthylisothiocyanate	D015058	551-06-4	DPYSL2	1808	1-Naphthylisothiocyanate results in increas	increases^expression	1	1
1-piperonylpiperazine	C078092	32231-06-	DPYSL2	1808	1-piperonylpiperazine results in increased	increases^expression	1	1
2,2',4,4',5-brominated dip	C477694		DPYSL2	1808	2,2',4,4',5-brominated diphenyl ether result	increases^expression	1	1
2,3,4,5-Tetrahydro-7,8-di	D015647	67287-49-	DPYSL2	1808	2,3,4,5-Tetrahydro-7,8-dihydroxy-1-phenyl	increases^expression	1	1
4-(5-benzo(1,3)dioxol-5-y	C459179		DPYSL2	1808	[NOG protein co-treated with trichostatin A	affects^cotreatment|decreases^expression	1	1
4-hydroxy-2-nonenal	C027576	29343-52-	DPYSL2	1808	4-hydroxy-2-nonenal binds to DPYSL2 pro	affects^binding	1	1
acetamide	C030686	60-35-5	DPYSL2	1808	acetamide results in increased expression	increases^expression	1	1
Acetaminophen	D000082	103-90-2	DPYSL2	1808	Acetaminophen results in increased expres	increases^expression	1	1
Acetaminophen	D000082	103-90-2	DPYSL2	1808	Acetaminophen results in increased expres	increases^expression	1	1
Aflatoxin B1	D016604	1162-65-8	DPYSL2	1808	Aflatoxin B1 results in increased methylati	increases^methylation	1	1
Albendazole	D015766	54965-21-	DPYSL2	1808	[Ivermectin co-treated with Albendazole] r	affects^cotreatment|increases^expression	1	1
Ammonium Chloride	D000643	12125-02-	DPYSL2	1808	Ammonium Chloride affects the expressior	affects^expression	1	1
Arsenic Trioxide	D0000772	1327-53-3	DPYSL2	1808	Arsenic Trioxide affects the expression of I	affects^expression	1	1
Arsenic Trioxide	D0000772	1327-53-3	DPYSL2	1808	Arsenic Trioxide results in increased expre	increases^expression	1	1
Atrazine	D001280	1912-24-9	DPYSL2	1808	Atrazine affects the methylation of DPYSL	affects^methylation	1	1
Benzo(a)pyrene	D001564	50-32-8	DPYSL2	1808	AHR protein affects the reaction [Benzo(a)	affects^expression|affects^reaction	1	1
Benzo(a)pyrene	D001564	50-32-8	DPYSL2	1808	Benzo(a)pyrene affects the methylation of	affects^methylation	1	1
Benzo(a)pyrene	D001564	50-32-8	DPYSL2	1808	Benzo(a)pyrene affects the methylation of	affects^methylation	1	1
Benzo(a)pyrene	D001564	50-32-8	DPYSL2	1808	Benzo(a)pyrene results in increased expres	increases^expression	2	1
benzo(e)pyrene	C026487	192-97-2	DPYSL2	1808	benzo(e)pyrene results in increased methy	increases^methylation	1	1
BEP protocol	C038328		DPYSL2	1808	BEP protocol results in increased expressi	increases^expression	1	1
bisphenol A	C006780	1980/5/7	DPYSL2	1808	bisphenol A affects the expression of DPY	affects^expression	2	2
bisphenol A	C006780	1980/5/7	DPYSL2	1808	[bisphenol A co-treated with Diethylhexyl F	affects^cotreatment|affects^methylation	1	1
bisphenol A	C006780	1980/5/7	DPYSL2	1808	bisphenol A results in increased expressior	increases^expression	1	1
bis(tri-n-butyltin)oxide	C005961		DPYSL2	1808	bis(tri-n-butyltin)oxide results in increasec	increases^phosphorylation	1	1
Carbamazepine	D002220	298-46-4	DPYSL2	1808	Carbamazepine affects the expression of D	affects^expression	1	1
Cisplatin	D002945	15663-27-	DPYSL2	1808	Cisplatin results in increased expression of	increases^expression	1	1
Clobetasol	D002990	25122-41-	DPYSL2	1808	Clobetasol results in increased expression	increases^expression	1	1
Clofibrate	D002994	637-07-0	DPYSL2	1808	Clofibrate affects the expression of DPYSL	affects^expression	1	1
Cocaine	D003042	50-36-2	DPYSL2	1808	[Cocaine affects the activity of PRKACA pr	affects^activity|affects^phosphorylation	1	1
cupric chloride	C029892	7447-39-4	DPYSL2	1808	cupric chloride results in increased expres	increases^expression	1	1
Dexmedetomidine	D020927	113775-47	DPYSL2	1808	Dexmedetomidine inhibits the reaction [Pr	decreases^reaction|increases^phosphorylatior	1	1
Dibutyl Phthalate	D003993	84-74-2	DPYSL2	1808	[bisphenol A co-treated with Diethylhexyl F	affects^cotreatment|affects^methylation	1	1
Diclofenac	D004008	15307-86-	DPYSL2	1808	Diclofenac affects the expression of DPYS	affects^expression	1	1
Diethylhexyl Phthalate	D004051	117-81-7	DPYSL2	1808	[bisphenol A co-treated with Diethylhexyl F	affects^cotreatment|affects^methylation	1	1
Diethylnitrosamine	D004052	55-18-5	DPYSL2	1808	Diethylnitrosamine results in increased exp	increases^expression	1	1
dihydroartemisinin	C039060		DPYSL2	1808	dihydroartemisinin analog binds to DPYSL	affects^binding	1	1
Ditiocarb	D004050	147-84-2	DPYSL2	1808	Ditiocarb results in increased expression o	increases^expression	1	1
dorsomorphin	C516138		DPYSL2	1808	[NOG protein co-treated with trichostatin A	affects^cotreatment|decreases^expression	1	1
Estradiol	D004958	50-28-2	DPYSL2	1808	Estradiol results in increased phosphorylat	increases^phosphorylation	1	1
Ethanol	D000431	64-17-5	DPYSL2	1808	[[Gasoline co-treated with Ethanol] results	affects^cotreatment|decreases^expression|inc	1	1
Finasteride	D018120	98319-26-	DPYSL2	1808	Finasteride results in increased expression	increases^expression	1	1
furan	C039281	110-00-9	DPYSL2	1808	furan results in increased expression of DF	increases^expression	1	1
Gasoline	D005742		DPYSL2	1808	[[Gasoline co-treated with Ethanol] results	affects^cotreatment|decreases^expression|inc	1	1
Gasoline	D005742		DPYSL2	1808	[Gasoline results in increased abundance c	affects^cotreatment|decreases^expression|inc	1	1
Gentamicins	D005839		DPYSL2	1808	Gentamicins results in increased expressio	increases^expression	1	1
Gentamicins	D005839		DPYSL2	1808	Gentamicins results in increased expressio	increases^expression	1	1
ICG 001	C492448		DPYSL2	1808	ICG 001 results in increased expression of	increases^expression	1	1
Indolinone A	C503753		DPYSL2	1808	Indolinone A results in increased phosphor	increases^phosphorylation	1	1
Isoflavones	D007529		DPYSL2	1808	Isoflavones results in increased expression	increases^expression	1	1
Isotretinoin	D015474	4759-48-2	DPYSL2	1808	Isotretinoin results in increased expression	increases^expression	1	1
Ivermectin	D007559	70288-86-	DPYSL2	1808	[Ivermectin co-treated with Albendazole] r	affects^cotreatment|increases^expression	1	1
jinfukang	C544151		DPYSL2	1808	jinfukang results in increased expression o	increases^expression	1	1
lead acetate	C008261	301-04-2	DPYSL2	1808	lead acetate results in increased expressio	increases^expression	1	1
Lovastatin	D008148	75330-75-	DPYSL2	1808	Lovastatin results in increased expression	increases^expression	1	1
Mercuric Chloride	D008627	7487-94-7	DPYSL2	1808	Mercuric Chloride affects the expression o	affects^expression	1	1
Methamphetamine	D008694	537-46-2	DPYSL2	1808	Methamphetamine affects the expression c	affects^expression	1	1
Methapyrilene	D008701	91-80-5	DPYSL2	1808	Methapyrilene results in increased methyla	increases^methylation	1	1
methylselenic acid	C008493		DPYSL2	1808	methylselenic acid results in increased exp	increases^expression	1	1
Methyltestosterone	D008777	58-18-4	DPYSL2	1808	Methyltestosterone results in increased ex	increases^expression	1	1
Miconazole	D008825	22916-47-	DPYSL2	1808	Miconazole results in increased expression	increases^expression	1	1
Morphine	D009020	57-27-2	DPYSL2	1808	Morphine deficiency affects the reaction [M	affects^reaction|decreases^expression	1	1
naphthalene	C031721	91-20-3	DPYSL2	1808	[naphthalene co-treated with CFTR gene n	affects^cotreatment|decreases^expression	1	1
N-benzylpiperazine	C461488		DPYSL2	1808	N-benzylpiperazine results in increased ex	increases^expression	1	1
O,O-diethyl O-3,5,6-trichl	C009618	5598-15-2	DPYSL2	1808	PON1 gene polymorphism affects the susc	affects^expression|affects^response to substa	1	1
Oxygen	D010100	7782-44-7	DPYSL2	1808	[NFE2L2 protein affects the susceptibility t	affects^expression|affects^response to substa	1	1
Particulate Matter	D052638		DPYSL2	1808	[[Gasoline co-treated with Ethanol] results	affects^cotreatment|decreases^expression|inc	1	1
Particulate Matter	D052638		DPYSL2	1808	[Gasoline results in increased abundance c	affects^cotreatment|decreases^expression|inc	1	1
pentabromodiphenyl ethe	C086401	32534-81-	DPYSL2	1808	pentabromodiphenyl ether results in increa	increases^expression	1	1
Pilocarpine	D010862	92-13-7	DPYSL2	1808	Pilocarpine results in increased expression	increases^expression	1	1
Plant Extracts	D010936		DPYSL2	1808	Plant Extracts results in increased express	increases^expression	1	1
Polycyclic Aromatic Hydro	D011084		DPYSL2	1808	[[Gasoline co-treated with Ethanol] results	affects^cotreatment|decreases^expression|inc	1	1
Polycyclic Aromatic Hydro	D011084		DPYSL2	1808	[Gasoline results in increased abundance c	affects^cotreatment|decreases^expression|inc	1	1
Potassium Dichromate	D011192	7778-50-9	DPYSL2	1808	Potassium Dichromate results in increased	increases^expression	1	1
Pravastatin	D017035	81093-37-	DPYSL2	1808	Pravastatin results in increased expression	increases^expression	1	2
Propofol	D015742	2078-54-8	DPYSL2	1808	Dexmedetomidine inhibits the reaction [Pr	decreases^reaction|increases^phosphorylatior	1	1
Propofol	D015742	2078-54-8	DPYSL2	1808	Propofol results in increased phosphorylati	increases^phosphorylation	1	1
Silicon Dioxide	D012822	7631-86-9	DPYSL2	1808	Silicon Dioxide results in increased expres	increases^expression	1	1
Sodium Dodecyl Sulfate	D012967	151-21-3	DPYSL2	1808	Sodium Dodecyl Sulfate results in increase	increases^expression	1	1
squalestatin 1	C075117		DPYSL2	1808	squalestatin 1 results in increased express	increases^expression	1	2
tetrabromobisphenol A	C020806	79-94-7	DPYSL2	1808	tetrabromobisphenol A results in increasec	increases^expression	1	1
Tetrachlorodibenzodioxin	D013749	1746-01-6	DPYSL2	1808	Tetrachlorodibenzodioxin affects the expre	affects^expression	1	1
Thioacetamide	D013853	62-55-5	DPYSL2	1808	Thioacetamide results in increased expres	increases^expression	1	1
titanium dioxide	C009495	13463-67-	DPYSL2	1808	titanium dioxide analog results in increase	increases^expression	1	1
Tretinoin	D014212	302-79-4	DPYSL2	1808	Tretinoin inhibits the reaction [PAX3 protei	affects^binding|decreases^reaction	1	1
Tretinoin	D014212	302-79-4	DPYSL2	1808	Tretinoin inhibits the reaction [TFAP2A pro	affects^binding|decreases^reaction	1	1
trichostatin A	C012589	58880-19-	DPYSL2	1808	[NOG protein co-treated with trichostatin A	affects^cotreatment|decreases^expression	1	1
Vanadates	D014638		DPYSL2	1808	Vanadates results in increased expression	increases^expression	1	1
Warfarin	D014859	81-81-2	DPYSL2	1808	Warfarin results in increased expression of	increases^expression	1	1
